# Current Sampling Methods for Gut Microbiota: A Call for More Precise Devices

**DOI:** 10.3389/fcimb.2020.00151

**Published:** 2020-04-09

**Authors:** Qiang Tang, Ge Jin, Gang Wang, Tianyu Liu, Xiang Liu, Bangmao Wang, Hailong Cao

**Affiliations:** ^1^Department of Gastroenterology and Hepatology, General Hospital, Tianjin Medical University, Tianjin, China; ^2^Tianjin Institute of Digestive Disease, General Hospital, Tianjin Medical University, Tianjin, China

**Keywords:** gut microbiota, sampling methods, feces, biopsy, aspirate

## Abstract

The development of next-generation sequencing technology has enabled researchers to explore and understand the gut microbiome from a broader and deeper perspective. However, the results of different studies on gut microbiota are highly variable even in the same disease, which makes it difficult to guide clinical diagnosis and treatment. The ideal sampling method should be non-invasive, involve little cross-contamination or bowel preparation, and collect gut microbiota at different sites. Currently, sequencing technologies are usually based on samples collected from feces, mucosal biopsy, intestinal fluid, etc. However, different parts of the gastrointestinal tract possess various physiological characteristics that are essential for particular species of living microbiota. Moreover, current sampling methods are somewhat defective. For example, fecal samples are just a proxy for intestinal microbiota, while biopsies are invasive for patients and not suitable for healthy controls. In this review, we summarize the current sampling methods and their advantages and shortcomings. New sampling technologies, such as the Brisbane Aseptic Biopsy Device and the intelligent capsule, are also mentioned to inspire the development of future precise description methods of the gut microbiome.

## Introduction

Humans harbor a complex gut microbiota whose composition varies between different regions in the gastrointestinal (GI) tract (Zoetendal et al., 2012). It has been reported that the number of uncultured species in the gut microbiota reached 1952 (Almeida et al., 2019). Physiological changes in different areas of the small intestine and colon, including chemical and nutritional gradients and isolated host immune activity, are thought to affect the composition of bacterial communities (Donaldson et al., 2016). The gut microbiota plays a critical role in the human internal environment. It evolves with the host and performs essential physiological functions for the host, such as preventing infection from various pathogens; promoting the maturation of the immune system; participating in the regulation of the immune response, nutritional absorption and metabolism; and promoting anti-cancer functions (Foster et al., 2017; Kim et al., 2017; Macpherson et al., 2017; Li et al., 2019). The colonization of newborn microbiota begins *in utero* (Dunn et al., 2017). Both the delivery mode and the cessation of breastfeeding are considered to be essential for adult-like gut microbiota assembly. The microbial composition changes abruptly during the 1st year of life (La Rosa et al., 2014; Bäckhed et al., 2015).

The gut microbiota changes gradually with time, and differences have been found between younger and older adults (O'Toole and Jeffery, 2015). The gut microbiota differs between individuals due to many factors, such as genes and diet. Studies have shown that high-carbohydrate and high-fiber diets could increase the abundance and diversity of intestinal microorganisms, especially in individuals with reduced microbial diversity (Tap et al., 2015; Sheflin et al., 2017). Low-carbohydrate diets can significantly reduce the number of butyric-acid-producing bacteria (such as *Roseburia* and *Bifidobacterium*), thereby reducing the production of butyric acid and reducing the protective effect on the intestine (Duncan et al., 2007; Russell et al., 2011). The immature gut microbiota is considered to be one of the causes of malnutrition, and human milk oligosaccharides can ameliorate malnutrition by regulating the microbiome (Blanton et al., 2016; Charbonneau et al., 2016). Moreover, the occurrence of many diseases, such as *Clostridium difficile* infection, inflammatory bowel disease (IBD) and irritable bowel syndrome (IBS), is also related to an alteration of gut microbiota. Long-term use of a large number of broad-spectrum antibiotics can lead to dysbiosis, such as *C. difficile* infection (Stanley and Burns, 2010). Compared with the control group, studies of intestinal microflora in IBD patients have consistently shown changes in microflora composition and reduced overall biodiversity, for instance, an increase in facultative anaerobes and a decrease in obligate anaerobes (Shim, 2013; Lloyd-Price et al., 2019). The occurrence of IBS is thought to be associated with the microbial effect on gut-brain communication (Eisenstein, 2016).

As there are numerous associations between gut microbiota and human health, it is particularly important to analyze the relationship between changes in gut microbiota and disease occurrence, progression, and prognosis. In the past, gut microbiome analysis depended on the isolation and cultures, but the difficulty in cultivating anaerobic bacteria, which are abundant in the intestine, seriously affected the accuracy of the analysis. In recent years, the progression of next-generation sequencing (NGS), which can accurately analyze microbial components without culture, has attracted attention in research on the intestinal microbiome. However, it is critical to collect appropriate samples of gut microbiota for NGS. Current sampling methods for obtaining specimens from feces, mucosal biopsy, and intestinal aspiration, all of which may have some defects, cannot accurately reflect the composition of the intestinal microbiome (Table 1). In this review, we summarize current methods for the collection of gut microbiota and their possible deficiencies to explore the difficulties that need to be overcome in gut microbiota collection technologies.

**Table 1:** Comparison of different sampling methods for gut microbiota analysis.

**Methods**	**Advantages**	**Disadvantages**
**Feces** 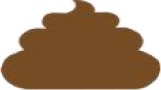	Convenient and repeatable sampling; non-invasive; sufficient biomass for analysis; inexpensive	Cannot accurately reveal the changes of gut microbiota; uneven distribution of bacteria within feces result in basis when homogenizing fecal samples
**Biopsy** 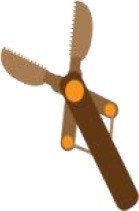	Accurate description of microbiota associated with tissue; controllable sampling site	Bowel preparation effects;invasive; inevitable contamination; insufficient biomass yield; expensive and time-consuming; not suitable for healthy control
**Luminal brush** 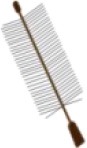	Accurate description of microbiota associated with tissue; controllable sampling site	Bowel preparation effects; invasive; inevitable contamination; expensive and time-consuming
**Laser capture microdissection** 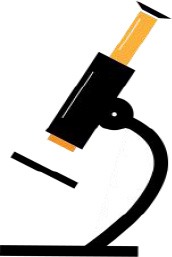	Accurately reflect host–microbe interactions; controllable sampling site	Bowel preparation effects; invasive; inevitable contamination; insufficient biomass yield; expensive and time-consuming; not suitable for healthy control
**Catheter aspiration** 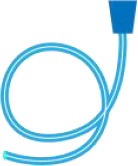	Accurate description of luminal microbiota; controllable sampling site	Bowel preparation effects; invasive; inevitable contamination; time-consuming; patient discomfort
**Intelligent capsule** 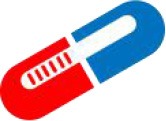	Accurate description of luminal microbiota; non-invasive; non-bowel preparation; no contamination	Expensive; great technical difficulty
**Surgery** 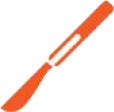	Accurate description of microbiota in sampling site; controllable sampling site; no contamination	Preoperative preparation effects; not suitable for healthy control
***in vivo*** **model (patients underwent ileostomy)** 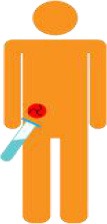	No contamination; convenient and repeatable sampling; non-invasive; sufficient biomass for analysis; inexpensive	No healthy control; Abnormal intestinal anatomy
**Fish** 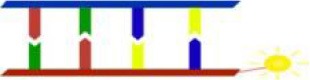	Accurately reflect spatial organization of microbiota and host–microbe interactions	Probe needs to be designed in advance; not suitable for complex microbiome

## Review

### Samples From Feces

For pragmatic reasons, fecal specimens are frequently used as proxies for gut microbiota. Fecal specimens are naturally collected, non-invasive and can be sampled repeatedly, so they are the source of samples for most intestinal microbiota studies. However, it is becoming increasingly clear that there may be significant differences in microbial composition between mucosa and feces (Zoetendal et al., 2002; Carroll et al., 2010). Feces were deemed to be substitutes for GI lumen contents, but their components uncertainly reflect direct interaction with mucosa. In recent studies, it has been demonstrated that the fecal and mucosal-associated microbiota are two distinct microbial niches (Rangel et al., 2015; Ringel et al., 2015; Tap et al., 2017). Fecal samples could not be indicators of the composition and metagenomic function of mucosa-associated microbiota distributed among multiple sites of the intestine (Zmora et al., 2018). Therefore, there is a bias in the estimation of intestinal microbiota with feces. Moreover, the fecal microbiota is not equally distributed within feces and has its own biostructure (Swidsinski et al., 2008). Wu et al. reported that 35% of low-abundance taxa, which account for 0.2–0.4% of the total microbiome in one replicate, were not found in a second fecal sample (Wu et al., 2010). The intraindividual variation in the detected bacteria was significantly reduced in the majority of studies that homogenized fecal samples or smears and ignored their structure (Hsieh et al., 2016). In the case of fecal subsampling, the results of microbial taxa detected by qPCR were highly variable (Gorzelak et al., 2015).

Additionally, under certain conditions, fresh stool samples cannot be analyzed immediately and need to be preserved for a while. Fecal materials instantly frozen at −80°C that can maintain microbial integrity without preservatives have been widely regarded as the gold standard for gut microbiota profiling. This approach retains microbial components similar to those of fresh samples and refrains from the potential impact of preservatives (Fouhy et al., 2015). For large-scale population studies, appropriate methods are important for patient compliance and the collection of optimal samples. Sometimes the ideal condition for immediate storage of specimens at −80°C cannot be met. Therefore, valid collection methods must be considered to minimize systematic bias that can be introduced in preprocessing steps (Flores et al., 2015). Jocelyn M et al. reported that the storage and transportation of samples at 4°C can minimize the changes to the microbial composition if ultralow-temperature storage is unavailable (Choo et al., 2015).

There are other storage methods with or without preservatives that are utilized to achieve microbiome compositions similar to those of fresh samples. As no-additive methods, fecal samples stored at room temperature for 24 h, −20°C for 1 week and in Eppendorf tubes at room temperature for 3 days did not significantly influence fecal microbiome profiles (Carroll et al., 2012; Tedjo et al., 2015). Additionally, fecal occult blood test cards, FTA cards (Whatman), and the OMNIgene Gut kit (DNA Genotek) have also been proven to be effective for samples stored for several days at room temperature (Dominianni et al., 2014; Song et al., 2016; Vogtmann et al., 2017). For using preservatives to store fecal specimens stably, 95% ethanol and RNAlater are worthy of recommendation (Flores et al., 2015; Song et al., 2016; Vogtmann et al., 2017; Wang et al., 2018). Storage conditions may significantly alter the characteristics of the microbial community. In the absence of ultralow-temperature conditions, storage and transportation by the methods mentioned above can minimize changes in microbial composition. The choice of collection and storage methods must be based on the purpose, scope, and conditions of the study.

In brief, the drawbacks of using fecal samples as substitutions for gut microbiota can be summarized in the following aspects. First, the possibility of incomplete separation of fecal bacteria and intestinal flora cannot be eliminated. Physiological variations containing chemical and nutrient gradients and the division of host immune activity are different among the lengths of the small and large intestine, all of which are known to influence the microbial composition. The families *Lactobacillaceae* and *Enterobacteriaceae* predominate in the small intestine, whereas the colon is dominated by the families *Prevotellaceae, Bacteroidaceae, Rikenellaceae, Ruminococcaceae*, and *Lachnospiraceae* (Donaldson et al., 2016). Therefore, it is not comprehensive to study intestinal flora with fecal bacteria. Second, homogenization before the collection of fecal samples perturbs fecal biostructure, and if not homogenizing, representativeness of samples may be inadequate. Swidsinski et al. used a plastic drinking straw to punch the stool to obtain fecal cylinders that successfully retained the biostructure of fecal microbiota and demonstrated that fecal microbiota is highly structured (Swidsinski et al., 2008). However, another study has reported that homogenization can significantly reduce the intraindividual variation in the detection of each fecal microbiota component (Hsieh et al., 2016). This leads to a controversy over which method should be adopted. Finally, in most cases, it is unrealistic to analyze fresh samples immediately. Then, the effect of the storage method, which may cause microbial DNA degradation, overgrowth, and the death of some species, on the fecal sample components must be considered.

## Samples From Endoscopy

Compared with the use of fecal samples to analyze the composition of the GI microbiota, few studies have been conducted to collect tissue samples and luminal contents to assess microbiota in different microbial niches during endoscopic procedures. More comprehensive information on the gut microbiome can be obtained by using tools (such as biopsy forceps and luminal brushes) through endoscopy. There are several common defects of sampling methods. First, endoscopy is invasive and not friendly to patients. Second, many studies have reported that the effect of bowel preparation on gut microflora is unavoidable. Then, when sampling tools go through the endoscopic channel, they may be contaminated by the content existing in the channel. Finally, because of the complex structure, endoscopy is limited to reach the distal small intestine. At present, there are several methods to obtain gut microbiota samples by endoscopy.

### Biopsy

The lower GI tract of mammals contains diverse microbial habitats along the small intestine, cecum, and colon. Endoscopic biopsy provides a way to investigate mucosal microbiota composition in different anatomical sites of the GI tract. The mucosal microbiota is thought to be important to the host because they are in contact with intestinal-related lymphoid tissue (Heinsen et al., 2015).

A bowel preparation usually requires a quantity of laxatives, such as polyethylene glycol (PEG) or sulfate, to clean out most of the digesta from the GI tract. Adequate bowel preparation requires the feces to present clear liquid without any solid particles within it. In mice with osmotic diarrhea induced by PEG aqueous solution, however, the intestinal epithelium, mucosa and gut environment of the host were destroyed in a short period of time, and the gut microbiota was still significantly changed for a long period of time. The changes in gut microbiota mainly included that the alpha diversity significantly decreased, and it was still significantly lower than the baseline level 2 weeks after diarrhea and was difficult to completely recover. Moreover, some high-abundance bacteria disappeared (e.g., the S24-7 family) and were replaced by other low-abundance taxa (Tropini et al., 2018). A previous study demonstrated that bowel preparation with PEG has the potential to result in significant morphological alterations in the colon, including the loss of epithelial cells and superficial mucus (Bucher et al., 2006). Shobar et al. reported that the diversity and composition of the luminal and mucosal microbiomes are affected by bowel preparation (Shobar et al., 2016). It has also been found that lavage before colonoscopy causes a 31-fold reduction in the total microbial load and the loss of the subject specificity of the microbiota in 22% of the participants (Jalanka et al., 2015).

In addition to the effects induced by bowel preparation, mucosal biopsies performed during standard endoscopic procedures may be contaminated by GI luminal fluid in the endoscopic channel. To minimize contamination during the sampling of mucosa-associated microbiota, the Brisbane Aseptic Biopsy Device (BABD), which consists of sterile forceps covered by a sheath and sealed by a plug at the ends, has been developed. Biopsies obtained by standard forceps have a greater diversity of mucosa-associated microbiota than samples collected using BABD (Shanahan et al., 2016). Even so, contamination may still happen before sampling. When the endoscopy tube enters the sampling site from the mouth or anus, it is inevitable for bacteria located in non-sampling sites to be brought to the sampling site. Moreover, the endoscope cannot reach all segments of the whole intestine, such as the distal small intestine, so the biopsy sections are restricted. Modern multi-omics technologies require different starting materials, including DNA, RNA, and proteins, and biopsy may not yield enough material to cope with the demands of these technologies. For this reason, Watt et al. demonstrated that colonic lavage offers a sample type similar to that of biopsy and generates significantly higher DNA than that of biopsies, with median DNA yields of 48.5 and 1.95 μg for colonic lavage and biopsy, respectively (Watt et al., 2016).

Mucosal biopsy only covers a small surface area and may result in sampling deviation and inaccessibility of rare taxa if the microbial population distributes unevenly. Mucosal biopsy often contains a large amount of contaminated host DNA, which complicates metagenomic and other molecular analyses (Huse et al., 2014).

Due to the influence of bowel preparation and contamination during the procedure, invasion, the limitation of sampling sites, the risk of bleeding and infection and its unsuitability for healthy people, biopsy, although deemed to be the gold standard for the collection of mucosal microbiota, is not appropriate for future gut microbiota analysis.

### Luminal Brushing

In 1979, Wimberley and colleagues first used the protected specimen brush (PSB) technique to collect infectious samples from the lower respiratory tract by the fiberoptic bronchoscope (Wimberley et al., 1979). These brush specimens are not easily contaminated by the normal flora of the upper respiratory tract, which is of greater significance for the diagnosis of lower respiratory tract infection. In recent years, Lavelle et al. promoted and validated techniques for repeated assessment of colonic microbial population spatial variability by combining mucosal biopsy with the PSB technique, which is used to sample the lumen-associated microbiota (Lavelle et al., 2013). The PSB is a sterile disposable sheath brush with a distal plug at the top, which is sealed in the sheath when inserted and retracted through the colonoscopic channel. In contrast to biopsy, mucosal brushing can reduce the risks associated with mucosal biopsy (bleeding and infection) and provide a more representative sample of the mucosal surface, and brush sampling has obtained a relatively large ratio of bacterial to host DNA (Huse et al., 2014). Although it has been reported that alpha diversities of samples collected by BABD and the PSB technique are similar at the phylum level, the PSB technique provides samples with a higher proportion of bacterial gDNA (Shanahan et al., 2016). Another study, however, suggested that there were spatial variations between luminal and mucosal microbiota (Lavelle et al., 2015). As sampling applying PSB technology depends on endoscopy, this method has the same defects as biopsies, such as bowel preparation influence, inevitable contamination, and invasion.

### Laser Capture Microdissection

Laser capture microdissection (LCM) was developed to overcome the drawbacks of tissue microdissection techniques. LCM selectively adheres materials of interest to the thin transparent film over the tissue section by a pulse from the infrared laser (Emmert-Buck et al., 1996). Then, the thin film with the obtained tissue is removed from the slice and treated directly with DNA, RNA or enzyme buffer. Therefore, this ability to selectively transfer the small focal region of tissue or cell clusters to film can be used to obtain mucous gel layers on the surface of intestinal biopsy samples. Before LCM samples can be analyzed, frozen biopsy samples need to be cut into 10-micron sections and then put onto nuclease- and nucleic-acid-free membrane slides and air-dried overnight. To capture interfold microbes of the mouse colon with high precision, Nava et al. used LCM to find that microbes in the interfold region were significantly different from those in the central luminal compartment (Nava et al., 2011). Although the maximum size of the interfold region is ~100 μm, the high resolution of LCM of ~5 μm allows easy and accurate sampling. Differentiation was evident between the luminal and mucosal intervals by using LCM to capture specimens of the mucus gel layer from snap-frozen biopsy samples (Lavelle et al., 2015). The detectable bacterial load of UC patients measured by targeted LCM and qPCR was lower than that of the control group (Rowan et al., 2010). Thus, LCM provides an easy, precise, and efficient method to obtain the bacteria in the mucosal region for the analysis of host-mucosa-associated microbiota interactions. LCM may be suitable for precision medicine, but the tedious procedure limits its use in large-scale studies. What limits the accuracy of LCM may be that the source of the sample is from biopsy, which has its own drawbacks, largely nucleic acid degradation, e.g., RNA, and an insufficient sample amount.

## Samples From Aspirated Intestinal Fluid

For aspirating uncontaminated intestinal fluid, Shiner invented a stainless-steel capsule fitted with a cap at its distal end and a hollow connection at the proximal end (SHINER, 1963). The proximal end of the capsule is connected to the negative-pressure source through a tube. When reaching the sampling site, the negative-pressure suction results in the opening of the sampling channel of the capsule, and the surrounding fluid entered the capsule chamber. After aspiration, the capsule is closed again, and the collected samples are isolated from the external fluid. The advantage of this device is to prevent the collected samples from being contaminated by the contents of the GI tract at the non-sampling sites. Due to the complex structure, this method has not been widely utilized. After that, the progress of obtaining GI fluid was in the development of a specially manufactured double-lumen tube with multiple aspirating ports in various locations and a mercury-filled bag at its distal end (Kalser et al., 1966). Subjects swallowed the tube, and then aspirates were sucked by a sterile syringe when ports were located in the proper position (75 cm distal to the ligament of Treitz for jejunal aspirates and 75 cm proximal to the ileocecal valve for ileal aspirates). Belov et al. aspirated intestinal fluid through nasojejunal tubes inserted routinely for enteral feeding (Belov et al., 1999). However, intestinal viscous fluid and blockages in the tubing made the collection procedure difficult and time-consuming.

Currently, endoscopic aspiration is most frequently used to obtain intestinal fluid. The aspiration and culture of small intestinal fluid are usually supposed to be the gold standard for the diagnosis of small intestinal bacterial overgrowth, which is defined as ≥ 10^5^ colony-forming units per milliliter (CFU/mL) upon the culture of aspirated fluid (Khoshini et al., 2008; Grace et al., 2013; Erdogan et al., 2015). A recent study based on the culture of duodenal aspirate demonstrated that SIBO is associated with an overgrowth of anaerobes and that the microbial composition of the small intestine in symptomatic patients changed significantly, which was inconsistent with the results of aspiration culture (Saffouri et al., 2019).

The endoscopic working channel is easily contaminated by oral and GI contents. Rubber was used to cover the distal tip of the catheter to block the infiltration of intestinal fluid (Uno et al., 1998). Inspired by previous studies, Quintanilha et al. used a membrane of microfilm to protect the distal tip to avoid internal contamination (Quintanilha et al., 2007). As endoscopic biopsy is aggressive for healthy people, the suction of intestinal fluid has become an alternative option. Nevertheless, intestinal fluid suction is sometimes time-consuming, which increases the time of endoscopy and sometimes fails due to sparse intestinal fluid (Riordan et al., 1995). Although previous studies have made great efforts to minimize co-contamination during intestinal fluid suction, innate defects in endoscopic sampling are unavoidable, as mentioned above. Moreover, the uncertainty of sampling sites also poses challenges for obtaining reliable samples.

## Samples From Surgery

When it is difficult to reach the distal ileum in endoscopy, surgery provides us with a way to sample the distal ileum. Methods to obtain intestinal flora at surgery include direct needle aspiration or the biopsy of mucosal samples (Bentley et al., 1972; Corrodi et al., 1978; Thadepalli et al., 1979; Lavelle et al., 2015). Since surgical sampling is not susceptible to contamination, in theory, the samples obtained from this method best represent the gut microbiota. However, the reality is that several preparations must be performed before surgery. These preparations may include fasting, mechanical bowel cleansing, and antibiotic administration, all of which can disrupt microbiota (Antonopoulos et al., 2009; Ubeda and Pamer, 2012; Ferrer et al., 2014; Zarrinpar et al., 2014; Jalanka et al., 2015). In this context, Thadepalli et al. drew duodenal, jejunal and ileal fluid from patients with abdominal trauma requiring emergency laparotomy by needle aspiration to explore microbiota of the small intestine. None of the patients underwent routine preoperative preparation; therefore, these samples obtained without interference from preoperative preparation were in the ideal condition. In addition, sampling during an operation can also circumvent the problem of small intestinal inaccessibility by using *in vivo* model systems (Booijink et al., 2007). Patients who undergo ileostomy can be used as an *in vivo* model and provide ileostomy effluent to obtain gut microbiota (Go et al., 1988; Ala Aldeen and Barer, 1989). Zoetendal et al. demonstrated that common microbial components in the samples excreted by ileostomists (individuals without a colon) could also be found in the small intestine of healthy subjects by the application of phylogenetic microarray analyses (Zoetendal et al., 2012). Compared to the colonic microbiota, the microbiota in the ileal effluent is relatively unstable and less complicated and consists of different dominating phylotypes (Booijink et al., 2010). Moreover, *in vivo* models can also be used to explore the effects of diet on the intestinal flora. Jonsson et al. investigated the effect of high-fiber intake on segmented filamentous bacteria by collecting human ileostomy samples (Jonsson, 2013). In addition to the above methods, Haysahi et al. obtained samples of intestinal contents from autopsy and demonstrated a gradient distribution in the number of OTUs from the proximal to the distal end of the intestine (Hayashi et al., 2005). Although the *in vivo* model provides convenience for sampling at any time, surgery itself results in significant alterations in the composition of gut microbiota that persist for a long time (Guyton and Alverdy, 2017). The ileostomy changes the anatomical structure of the intestine that may have an irreversible effect on the composition of the gut microbiota. Therefore, it is not clear whether the research results based on ileostomy effluent are suitable for people with normal anatomical structures. As surgery is invasive, acquiring a sample from healthy controls seems impossible. For operation and autopsy, surgical application is obviously limited. Surgery is not conducive to a comprehensive analysis of the relationship between bacterial flora and diseases in different populations.

## Ingestible Sampling Devices

The drawbacks of the above methods seem to be insurmountable, and researchers are putting forth effort to develop new devices for sampling. To date, many swallowable devices have been used to observe the intestine and deliver drugs. Due to the non-invasive characteristics of swallowable devices, their use is increasingly considered to collect intestinal contents. Based on microelectromechanical systems (MEMS) technology, Cui et al. invented a swallowable capsule that can deliver drugs and collect intestinal fluid (Cui et al., 2008). The characteristics of gastrointestinal tract positioning, wireless communication and large sample size give the capsule the ability to automatically collect intestinal fluid. However, the limitation of this device is that the collected sample is easily polluted by the downstream liquid. In recent years, NIZO has developed an intelligent capsule for small intestinal sampling of the microbiomeby combining the IntelliCap® system and the quencher. The IntelliCap® system is a swallowable capsule that contains pH and temperature sensors, communication units, μ-computers, motors and batteries. The quencher is a container placed in the capsule for qualitative and quantitative preservation of microbiota. The capsules can be positioned by measuring significant changes in pH in the GI tract (Koziolek et al., 2015). When the swallowed capsule reaches the designated region of the small intestine, the aspiration of the intestinal fluid can be initiated. Aspirated intestinal fluid can be collected after the capsule is discharged from the body. Recently, Rezaei Nejad et al. also reported a 3D-printed pill for aspirating small intestinal fluid (Rezaei Nejad et al., 2019). This pill comprises a semipermeable membrane to separate the helical channels and salt chamber. The higher osmotic pressure on the side of the salt chamber drives the liquid in the helical channels to flow to the chamber through the semipermeable membrane. Then, the intestinal fluid can be aspirated from the inlets connected to the helical channels. The outer enteric capsule shell ensures that the collection begins in the small intestine. Compared to NIZO's capsule, the cost of this battery-less pill will certainly be much lower. However, the problem of sample preservation after collection seems to have not been solved, which may lead to the contamination of samples with intestinal fluid from non-collected sites.

Our present work focuses on the collection of intestinal fluid samples by minorly invasive methods. We also investigated an inexpensive and convenient capsule device, Intestine Microbiome Aspiration (IMBA), which aims to collect intestinal fluid samples autonomously. Without using expensive microelectromechanical system technologies, IMBA utilizes controlled-release technology equipped with a novel sampling mechanism to achieve precise and regional sampling in the intestine. Moreover, the form of capsules improves patient compliance, and sampling conditions closer to the physiological state (no need for bowel preparation) provide higher accuracy. The key of this technology is how to accurately locate and collect intestinal fluid.

## Biology-Related Tools

In addition to the composition and diversity of gut microbiota, their spatial organization also reflects the host-microbiota relationship. To obtain the complete structure of the intestine and its contents, Johansson et al. improved the histological preparations that successfully preserved intestinal mucus and located bacteria with fluorescence *in situ* hybridization (FISH) (Johansson and Hansson, 2012). Using FISH technology, the location of the bacteria of interest labeled by a fluorescent DNA probe can be observed under a fluorescence microscope. However, due to the difficulties in sampling, ethical problems and the huge individual differences in microbial composition, the research and manipulation of human gut microbial communities *in situ* are limited. As an alternative, the transplantation of human gut microbiota into germ-free mice has been widely used (Goodman et al., 2011; McNulty et al., 2013). To explore the spatial organization of the human gut microbiota, Earle et al. developed a new approach that visualizes the bacteria in human microbiota-colonized gnotobiotic mice by FISH (Earle et al., 2015). They inoculated the fluorescent probe corresponding to the bacteria of interest to the fixed gut cross-sections of mouse intestine. However, a single field of view in a section cannot represent the entire intestine. To solve this problem, Bacspace software was developed to stitch the overlapping images of multiple fields of view into a continuous image that represents the entire gut, distinguish host epithelial cells from bacteria and measure the distance between bacterial cells and between bacterial cells and the epithelium. Using Bacspace, they revealed homologous clustering within *Bacteroidales* or *Firmicutes*, which clusters of *Bacteroidales* that exclude *Firmicutes* and vice versa. Moreover, the application of FISH combined with spectral imaging analysis methods uncovered the spatial organization of gnotobiotic mice colonized with 15-member human gut microbiota (Welch et al., 2017). There are two densely colonized regions in the colon: one adjacent to the mucosa and the other bordered by food particles within the lumen. Modest differences in the composition of the microbiota in these two regions suggest that the lumen and mucosa should not be defined as stratified compartments. Owing to orders-of-magnitude differences in the microbial density between the small intestine and colon, the number of microbes in the cross-section of the small intestine is 10 to 1 million times less than that in the colon. That is, compared with 1,000 bacteria per field of view in the colon, there are almost no bacteria in the small intestine. Because of its higher microbial density, the histological method is more suitable for the colon. Improper sample preparation can also result in the loss of intestinal contents in sections. Compared with other methods, the Technovit h8100 embedding method can successfully preserve the three-dimensional structure of the intestine and is compatible with FISH and other labeling techniques for the visualization of microbial cells in the mouse intestines along with mucus and fecal pellets (Hasegawa et al., 2017). These imaging techniques can simultaneously locate some cultivable microbes with fluorescent probes that need predesign, but they cannot deal with complex and diverse microbiomes. For unbiased high-taxonomic-resolution dissection of the complex gut microbial biogeography, Ravi et al. developed metagenomic plot sampling by sequencing that can analyze the spatial location of various microbes without advance specification (Sheth et al., 2019). They found that a strong association between *Bacteroides* in all intestinal cavities and the local areas of bacterial phylogeny and aggregation were related to dietary disturbance. Although the establishment of human gut microbiota in germ-free mice provides us with a solution to the difficulty of sampling in the human intestine, the influence of the difference in gene background on the composition of microbiota should not be ignored (Wos-Oxley et al., 2012). At the same time, the establishment of human intestinal microflora in germ-free mice will also be affected by the operation of bacterial transplantation. There are several points to be paid attention to for oral gavage. Because of the presence of anaerobic bacteria in the human flora, they need to be rapidly infused into the digestive tract. A larger volume of administration will promote the distribution and colonization of the mouse intestinal microbial community and protect the microbiota from intestinal enzymes and pH changes, and some rodent diets may also promote or inhibit the growth of some bacteria (Rodriguez-Palacios et al., 2019) Compared with controls, germ-free mice have a significantly longer gut transit time along with lower levels of SCFAs that are produced by the fermentation of indigestible carbohydrates by commensal bacteria and can promote bowel movement (Vincent et al., 2018). Colonization of different strains in germ-free mice may affect intestinal motility by influencing the level of SCFAs, resulting in different amount of fecal pellets in the colon. Therefore, the relationship between the bacteria and fecal pellets in the colon section may be slightly different.

## Perspective

Despite considerable efforts by researchers to obtain accurate samples, the shortcomings of current sampling methods are bound to be insurmountable. It will be difficult to obtain accurate results from inaccurate samples. Feces have become the sample source of most bacterial flora studies because of their convenience and non-invasive nature, but even the microbiota content in the lower digestive tract, which is closest to feces, is significantly different from that of feces (Zmora et al., 2018). Most of the remaining sampling methods are invasive and not suitable for healthy people. Issues to be solved in future sampling methods should include reducing invasiveness, non-cross-contamination sampling at fixed points and minimizing disturbance to normal intestinal physiology.

The accuracy of samples has a remarkable effect on the value of studies of gut microbiota; therefore, more precise sampling methods are needed to ensure the reliability of research. The design of future optimal intestinal microbiota collection devices should accord with the following requirements. First, the devices can collect intestinal contents at a fixed point effectively and prevent the samples from cross-contamination. Second, the size of the devices must be small, which enables smooth passage through the pylorus and ileocecal valve. Then, the device is simple in structure and easy to operate, and the sampling process causes less psychological pressure and discomfort. Material used in manufacturing equipment should be non-toxic, harmless, non-teratogenic, and non-carcinogenic. Moreover, the cost of devices is also a pivotal consideration for large cohort studies. Finally, given that bowel preparation has a greater impact on the composition of gut microbiota, new technologies are best to eliminate this procedure. In view of the shortcomings of current sampling methods, the development of more accurate sampling methods is critical for future gut microbiota research. To meet these requirements, the development of swallowable devices seems to be the most feasible method. In the future, small, autonomous sampling swallowable devices will enable researchers and clinicians to study intestinal flora with specificity, localization and sensitivity. On the other hand, the spatial structure of intestinal flora is also an important component of studying the interaction between flora and host. For ethical reasons, it seems impractical to collect samples containing information on the positional relationship between the microbes and the gut. As an alternative, the establishment of human gut microbiota in gnotobiotic mice also provides us with a solution to the difficulties of sampling. Although fluorescence imaging cannot study complex microbiomes, the application of unbiased spatial macrogenomics in gnotobiotic mice will greatly promote our understanding of the spatial organization of gut microbiota.

## Author Contributions

QT, GJ, GW, BW, and HC designed the research. QT, GJ, GW, TL, and XL collected and analyzed relevant information. QT, GJ, GW, and HC wrote the paper. All authors were involved in the final approval of the article.

### Conflict of Interest

The authors declare that the research was conducted in the absence of any commercial or financial relationships that could be construed as a potential conflict of interest.
